# A linear sequence to facilitate curation of herbarium specimens of Annonaceae

**DOI:** 10.1007/s12225-018-9764-3

**Published:** 2018-08-14

**Authors:** Lars W. Chatrou, Ian M. Turner, Bente B. Klitgaard, Paul J. M. Maas, Timothy M. A. Utteridge

**Affiliations:** 1 Wageningen University and Research, Biosystematics group, Droevendaalsesteeg 1, 6708 PB Wageningen, The Netherlands; 2 Singapore Botanical Liaison Officer, Royal Botanic Gardens, Kew, Richmond, Surrey, TW9 3AB UK; 3 Herbarium, Singapore Botanic Gardens, National Parks Board, 1 Cluny Road, Singapore, 259569 Singapore; 4 Royal Botanic Gardens, Kew, Richmond, Surrey, TW9 3AB UK; 5 Naturalis Biodiversity Center, Section Botany, P.O. Box 9517, 2300 RA Leiden, The Netherlands

**Keywords:** Annonaceae, classification, herbaria, phylogenetic hypotheses, systematics, taxonomy

## Abstract

**Summary.** This paper provides a linear sequence of four subfamilies, 15 tribes and 106 genera of the magnoliid family Annonaceae, based on state-of-the-art and stable phylogenetic relationships. The linear sequence facilitates the organisation of Annonaceae herbarium specimens.

## Introduction

Plant taxonomy is a scientific expression for one of the defining characteristics of the human species: observing, assembling and classifying. The ordering of the plant world has been attempted ever since the origin of modern man, and even before. Neanderthals were able to distinguish fruit, nuts, roots, bulbs and tubers that were exploited as food resources (Henry *et al*. 2011), and upper palaeolithic hunter-gatherers recognised and categorised plants for economic and ritual uses (Nadel *et al*. 2013; Power *et al*. 2014). From Theophrastus onwards, in the fourth century BC, botanists have attempted to organise plants into classification systems. The sexual system published by Linnaeus (1753) in his *Systema Naturae* is the classical example of a classification system that has been designed for convenience, most notably to facilitate plant recognition and identification, and unequivocal communication about plants.

In Linnaeus’s time the practice of drying and conserving plants for future study was well established. In the first half of the 16^th^ century, the Bolognese botanist Luca Ghini introduced a new way of studying plants by making the earliest *hortus siccus*. By pressing plants and storing them in a book, he invented the herbarium. It is in this era that botanic gardens, illustrated botanical publications, and herbaria were established as a trinity of resources for botanical sciences, a foundation that is still fundamental to botanical research today.

Botany in the eighteenth and nineteenth century to a large extent involved the rejection of Linnaeus’ artificial system, replacing it with classifications that reflected supposed evolutionary relationships based on careful observations of plant characters. This endeavour was greatly facilitated by collections that arrived in Europe from all over the world and were kept in newly established flourishing herbaria. Up to that point, private ownership of plant collections had been common practice. In the mid-19^th^ century, however, these collections were often sold to the burgeoning herbaria, with the specific goal of making the collections available for study by staff and visitors. Until then, classifications such as those by Linnaeus and de Jussieu (1789) had primarily been based on European temperate plants. The influx of samples representing the wide plant diversity in colonial territories challenged these classification systems, with many non-temperate plant groups such as Annonaceae that were largely unknown to European botanists. Proliferating collections and botanical studies resulted in natural classifications by, e.g., Bentham & Hooker (1862 – 1883) and Engler & Gilg (1924), which have been the basis for taxonomic literature and for the arrangement of herbaria and botanic gardens for a long time. Given the Herculean task of changing the classification system followed in any sizeable herbarium (e.g. Wearn *et al*. 2013; Le Bras *et al*. 2017), many herbaria are still organised to date on the basis of outmoded classification systems going back in time a century or more.

Over the past two decades or so, phylogenetic systematics has resulted in a notable transformation of the classification of plants, and of angiosperms in particular. Based on the results of phylogenetic analyses, initially the delineation of angiosperm orders and families was evaluated and changed if necessary to make plant families comply with the prime guiding criterion of monophyly (APG I 1998; APG II 2003; APG III 2009; APG IV 2016). Subsequently, working groups of systematists have applied the results of phylogenetic analyses to revise infrafamilial classifications (e.g. Schneider *et al*. 2014; Bone *et al*. 2015; Chacón *et al*. 2016; Claudel *et al*. 2017; De Faria *et al*. 2017; Simões & Staples 2017), an endeavour that is still ongoing. Systematists have spent great effort in revising the classification of angiosperms because of the awareness that phylogenetics has brought methodological rigour to systematics and predictivity to classifications, which enabled the treatment of phylogenetic relationships — and therefore of classifications — as testable hypotheses, rather than opinions of scientists, however scholarly they might be.

Recently, the herbarium of the Royal Botanic Gardens, Kew, was reorganised following the APG III system at the family level and taking phylogenetic classifications into account at the infrafamiliar level. Linear sequences of plant taxa enable curators to curate herbarium collections in accordance with phylogenetic relationships among genera. Linear sequences reflect the order of names attached to the tips of a phylogenetic tree, after the branches in the tree have been ordered according to some projection method. Alternatively, herbarium collections may be organised alphabetically, and the choice between an arrangement based on alphabet or on classification has been cause for debate (Funk 2003; Burger 2004). Storing collections according to any organising system remains indispensable as herbaria have retained their historic functions, being the basis for plant systematics and taxonomy, floristics and identification, assessment of botanical diversity, and teaching. In addition, scientific developments have unlocked new applications of herbarium collections, such as the characterisation of phenological responses to climate change (Willis *et al*. 2017), the assessment of global rarity of plant species to guide conservation (bioquality; Marshall *et al*. 2016), the sequencing of near-complete plastomes (Bakker *et al*. 2016; Hoekstra *et al*. 2017) and the targeted enrichment of nuclear genes (Hart *et al*. 2016), both for phylogenetic and evolutionary studies.

In this paper, we present a linear sequence of genera of Annonaceae. Generally, the family is among the most species-rich and abundant families in tropical rain forest communities (e.g. Cardoso *et al*. 2017; Sosef *et al*. 2017; Turner in press) and is amply represented in major herbaria.

## Linear sequences

Haston *et al*. (2007, 2009) published a simple methodology for translating tree-like relationships into a linear sequence, and applied this to a phylogenetic tree of angiosperm families. Similarly, linear sequences have been produced for gymnosperms (Christenhusz *et al*. 2011a) and lycophytes and ferns (Christenhusz *et al*. 2011b). In order to extend the phylogenetic arrangement of collections to the level of genera, linear sequences that translate family phylogenies are indispensable. So far, linear sequences are available for Fabaceae (Lewis *et al*. 2013), and monocots excluding Poaceae and Orchidaceae (Trias-Blasi *et al*. 2015).

The assembly of the phylogenetic tree underpinning the linear sequence, and the translation of the tree into the sequence consisted of the following steps.

**Fig. 1:**
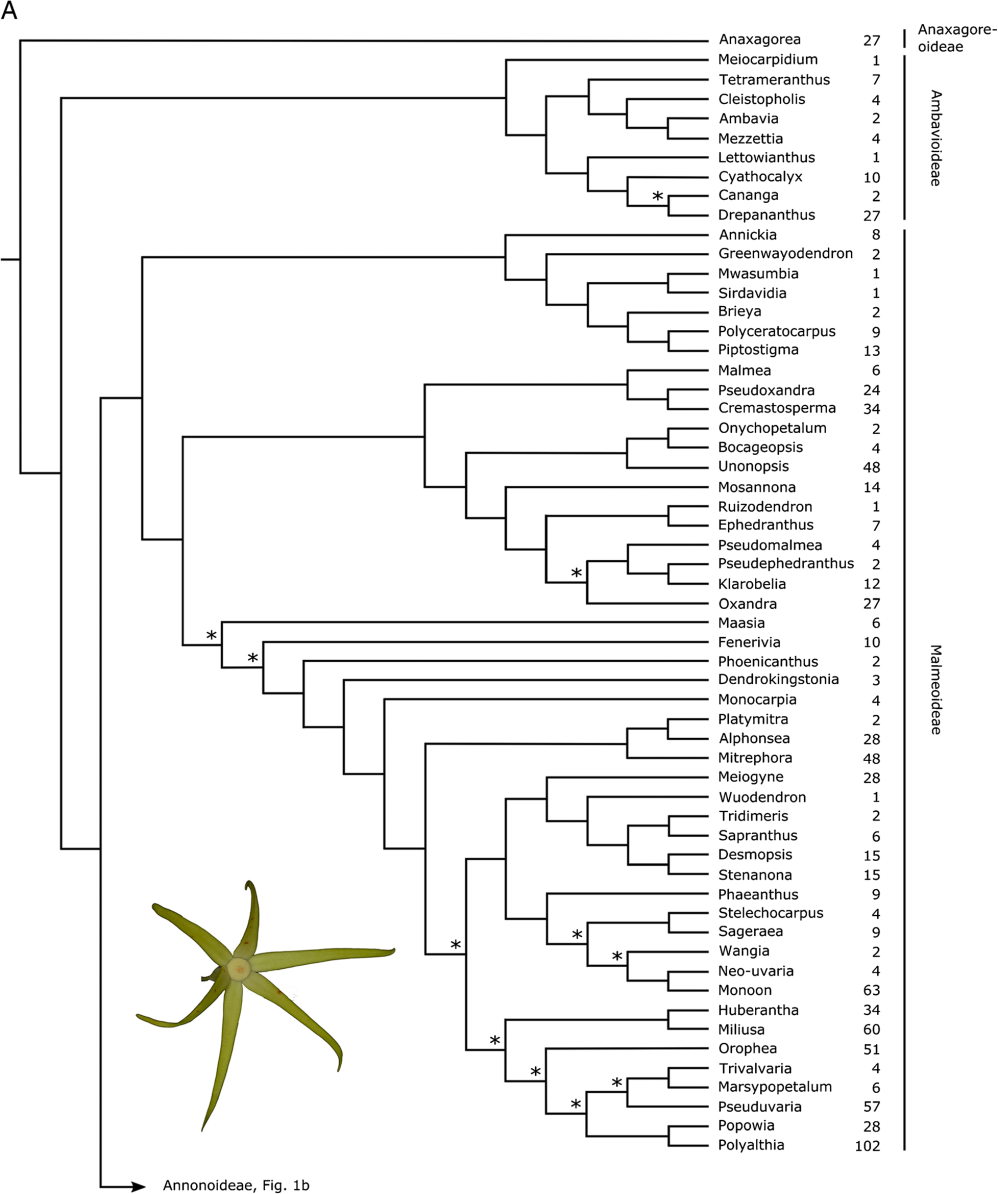

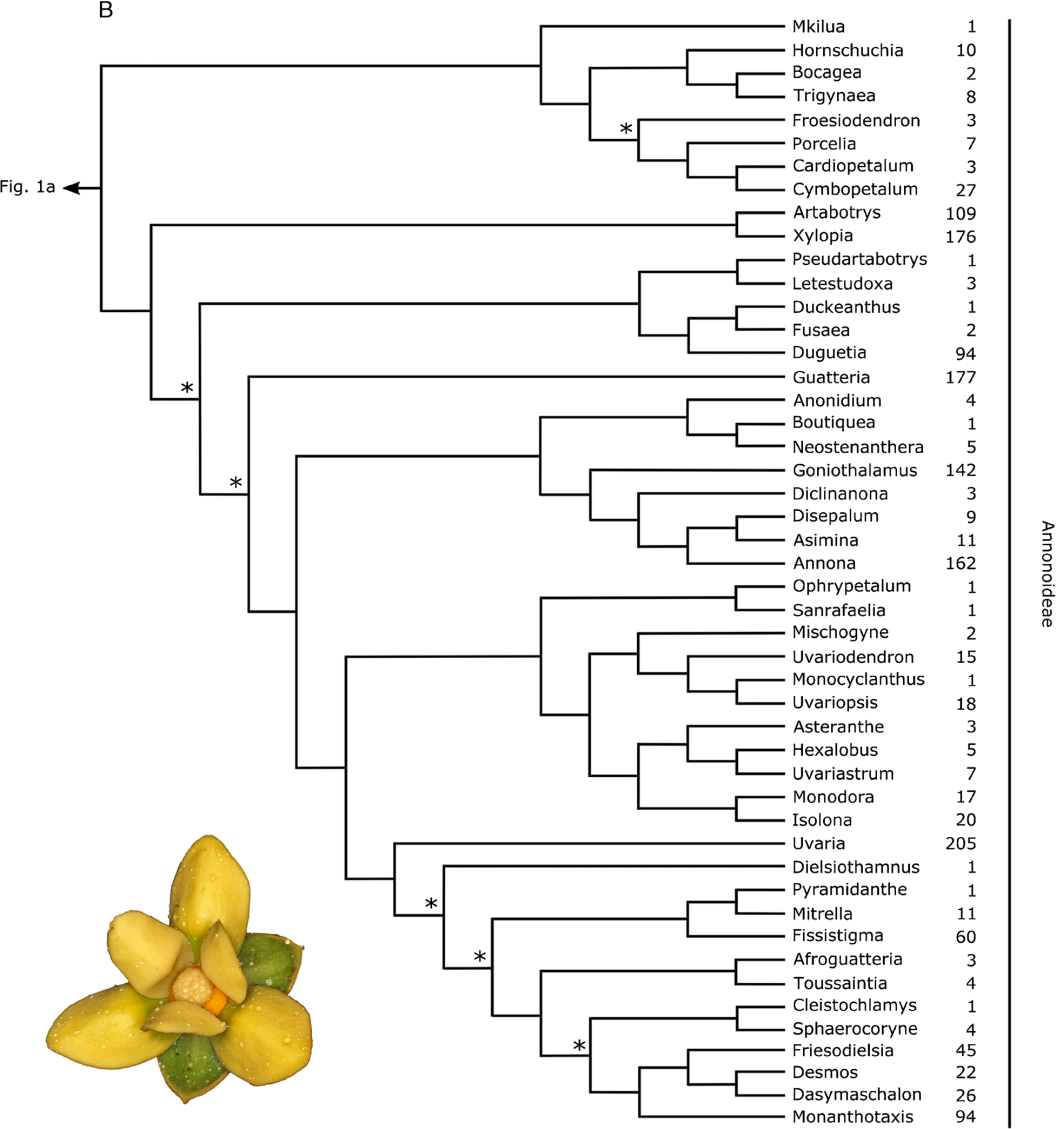
Summary tree underlying the linear sequence of Annonaceae genera (Fig. 1a: Anaxagoreoideae, Ambavioideae and Malmeoideae; Fig. 1b: Annonoideae). Nodes marked with an asterisk have not received significant support (parsimony or maximum likelihood bootstrap, Bayesian posterior probability) in any publication. The number of species for each genus is indicated. Inset pictures show flowers of *Fenerivia capuronii* (Cavaco & Keraudren) R. M. K. Saunders (Fig. 1a) and *Guatteria aeruginosa* Standl. (Fig. 1b). photos: l. w. chatrou.

- a summary tree showing relationships of all genera of Annonaceae was assembled. Details are given below, in the section ‘Annonaceae classification’. Nodes that did not receive significant support (parsimony or maximum likelihood bootstrap percentages, Bayesian posterior probabilities) in any of the published studies were resolved according to the topology most frequently inferred in all used publications.
- we defined clade size in terms of number of species, and not number of higher taxa (e.g. number of genera to define the size of tribes) as the former estimate of clade size can be expected to be more stable than the latter, i.e. more robust to changing taxonomic concepts (Hawthorne & Hughes 2008).
- species numbers for all genera were taken from Annonbase (Rainer & Chatrou 2006).
- following Haston *et al*. (2007), nodes of the phylogenetic tree were rotated in such a way that clades with fewer species were placed before clades with more species. This clade size criterion was applied subsequently to all nodes in the tree, starting from the root node (Fig. 1). The names along the tips, reading down from the top, represent the linear sequence.

## Annonaceae classification

Historically, botanists have been reluctant to provide a classification for genera in the magnoliid family Annonaceae. Even though subfamilies and tribes were described by eminent botanists such as Rafinesque (1815), Endlicher (1839), Hooker & Thomson (1855) and Baillon (1868), these were hardly used by Annonaceae workers at the end of the 20^th^ century, just before the breakthrough of phylogenetic methods. The classification most frequently referred to was the one by Fries (1959), who identified informal groups of genera but was reluctant to solidify his arrangement into a formal classification. Based on phylogenetic analyses of almost all genera, Chatrou *et al*. (2012) revised the infrafamilial classification of the family, and divided the family into four subfamilies and 14 tribes. An addition to the classification by Chatrou *et al*. (2012) was published by Guo *et al*. (2017b) with the new tribe Phoenicantheae, necessary to achieve monophyly of infrafamilial taxa belonging to the grade of species-poor lineages basal to tribe Miliuseae. Further detailed phylogenetic studies of specific tribes and genera, or the discovery of new genera of Annonaceae, did not reveal any previously overlooked deeper lineages within the family, and changes in generic circumscription could be accommodated into existing tribes (Chaowasku *et al*. 2012; Chaowasku *et al*. 2013; Guo *et al*. 2014; Xue *et al*. 2014; Chaowasku *et al*. 2015; Couvreur *et al*. 2015; Tang *et al*. 2015; Thomas *et al*. 2015; Ortiz-Rodriguez *et al*. 2016; Ghogue *et al*. 2017; Guo *et al*. 2017a; Stull *et al*. 2017; Pirie *et al*. 2018). Thus, we have arrived at a point where the classification of Annonaceae, principally based on plastid sequence data, can be considered stable, and can be used to arrange herbarium specimens. Annonaceae currently contain 2430 species (Rainer & Chatrou 2006, accessed 15th May 2018), classified into 106 genera.

A few decisions, which cannot be derived from the large-scale phylogenetic trees in Chatrou *et al*. (2012), Chaowasku *et al*. (2014) and Guo *et al*. (2017b), need to be justified:

- We consider the generic name *Haplostichanthus* a synonym of *Polyalthia*. Xue *et al*. (2012) reduced nine species of *Haplostichanthus* into synonymy of *Polyalthia*, but did not discuss the status of *Haplostichanthus gamopetala* (Boerl. ex Koord.) Heusden. Given the morphological similarities of this species with other former species of *Haplostichanthus* (van Heusden 1994a) we consider this species a synonym of *Polyalthia gamopetala* Boerl. ex Koord., thus removing the name *Haplostichanthus* from accepted names in Annonaceae taxonomy.
- Thomas *et al*. (2012) showed that species formerly included in *Oncodostigma* are nested in *Meiogyne*, thus validating the classification of some species of *Oncodostigma* in the latter genus by van Heusden (1994b). The last remaining species of *Oncodostigma* have recently been sunk into synonymy of *Meiogyne* (Turner & Utteridge 2015; Xue *et al*. 2017), making the further use of the name *Oncodostigma* unnecessary.
- We have not included the genus *Melodorum.* The type specimen of the type species *Melodorum fruticosum* Lour. cannot be distinguished from *Uvaria siamensis* (Scheff.) L. L. Zhou, Y. C. F. Su & R. M. K. Saunders. The problem has been that *Melodorum fruticosum* has widely been misapplied to *Sphaerocoryne* spp. Guo *et al*. (2017b) note this as the probable reason that the position of *Melodorum* was not resolved before.
- For the tribe Malmeeae we based the linear sequence on Chatrou *et al*. (2012) and Pirie *et al*. (2006) with one exception. Analysing an alignment of 66 plastid markers, Lopes *et al*. (2018) inferred the *Malmea* / *Cremastosperma* / *Pseudoxandra* clade as sister to the remaining Malmeeae, instead of the *Onychopetalum* / *Bocageopsis* / *Unonopsis* clade. As these relationships were maximally supported in Bayesian analyses we adopt the result of Lopes *et al*. (2018).
- the position of *Diclinanona* was clarified in Erkens *et al*. (2014), that of *Wangia* was taken from Guo *et al*. (2014).
- we consider the genus *Winitia* (Chaowasku *et al*. 2013) a synonym of *Stelechocarpus* (Turner 2016).

## Linear sequence of Annonaceae

Accepted names are listed in bold and synonyms in italics. We listed *Unona* L.f. and *Uva* Kuntze as synonyms of *Xylopia* and *Uvaria* respectively, as the type specimens of the former two genera have been put into synonymy of the latter two. Note, however, that species previously classified in *Unona* or *Uva* can now be found in dozens of genera of Annonaceae.

We considered it beyond the scope of this paper to include details on revisions and other taxonomic information, for which we refer to a recent overviews (e.g. Maas *et al*. 2011; Erkens *et al*. 2012) and continuously updated taxonomic data in Annonbase (Rainer & Chatrou 2006), and the website http://annonaceae.myspecies.info.

A naxagoreoideae C hatrou , P irie , E rkens & C ouvreur

**Anaxagorea** *A.St.-Hil.*

*Eburopetalum* Becc., *Pleuripetalum* T. Durand, *Rhopalocarpus* Teijsm. & Binn. ex Miq.

A mbavioideae C hatrou , P irie , E rkens & C ouvreur

**Meiocarpidium** *Engl. & Diels*

**Tetrameranthus** *R. E. Fr.*

**Cleistopholis** *Pierre ex Engl.*

**Ambavia** *Le Thomas*

**Mezzettia** *Becc.*

*Lonchomera* Hook. f. & Thomson

**Lettowianthus** *Diels*

**Cyathocalyx** *Champ. ex Hook. f. & Thomson*

**Cananga** (*Dunal*) *Hook. f. & Thomson*

*Canangium* Baill. ex King, *Fitzgeraldia* F. Muell.

**Drepananthus** *Maingay ex Hook. f.*

M almeoideae C hatrou , P irie , E rkens & C ouvreur

Piptostigmateae Chatrou & R. M. K. Saunders

**Annickia** *Setten & Maas*

*Enantia* Oliv.

**Greenwayodendron** *Verdc.*

**Mwasumbia** *Couvreur & D. M. Johnson*

**Sirdavidia** *Couvreur & Sauquet*

**Brieya** *De Wild.*

**Polyceratocarpus** *Engl. & Diels*

*Alphonseopsis* Baker f., *Dielsina* Kuntze

**Piptostigma** *Oliv.*

Malmeeae Chatrou & R. M. K. Saunders

**Malmea** *R. E. Fr.*

**Pseudoxandra** *R. E. Fr.*

**Cremastosperma** *R. E. Fr.*

**Onychopetalum** *R. E. Fr.*

**Bocageopsis** *R. E. Fr.*

**Unonopsis** *R. E. Fr.*

**Mosannona** *Chatrou*

**Ruizodendron** *R. E. Fr.*

**Ephedranthus** *S. Moore*

**Pseudomalmea** *Chatrou*

**Pseudephedranthus** *Aristeg.*

**Klarobelia** *Chatrou*

**Oxandra** *A. Rich.*

Maasieae Chatrou & R. M. K. Saunders

**Maasia** *Mols, Kessler & Rogstad*

Fenerivieae Chatrou & R. M. K. Saunders

**Fenerivia** *Diels*

Phoenicantheae X. Guo & R. M. K. Saunders

**Phoenicanthus** *Alston*

Dendrokingstonieae Chatrou & R. M. K. Saunders

**Dendrokingstonia** *Rauschert*

*Kingstonia* Hook. f. & Thomson

Monocarpieae Chatrou & R. M. K. Saunders

**Monocarpia** *Miq.*

Miliuseae Hook. f. & Thomson

**Platymitra** *Boerl.*

*Macanea* Blanco

**Alphonsea** *Hook. f. & Thomson*

**Mitrephora** (*Blume*) *Hook. f. & Thomson*

*Kinginda* Kuntze

**Meiogyne** *Miq.*

*Ancana* F. Muell., *Ararocarpus* Scheff., *Chieniodendron* Tsiang & P. T. Li, *Fitzalania* F. Muell., *Guamia* Merr., *Oncodostigma* Diels, *Polyaulax* Backer

**Wuodendron** *B. Xue, Y. H. Tan & Chaowasku*

**Tridimeris** *Baill.*

**Sapranthus** *Seem.*

**Desmopsis** *Saff.*

*Reedrollinsia* J. W. Walker

**Stenanona** *Standl.*

**Phaeanthus** *Hook. f. & Thomson*

**Stelechocarpus** *Hook. f. & Thomson*

*Winitia* Chaowasku

**Sageraea** *Dalzell*

**Wangia** *X. Guo & R. M. K. Saunders*

**Neouvaria** *Airy Shaw*

**Monoon** *Miq.*

*Cleistopetalum* H. Okada, *Enicosanthum* Becc., *Griffithia* Maingay ex King, *Griffithianthus* Merr., *Marcuccia* Becc., *Woodiella* Merr., *Woodiellantha* Rauschert

**Huberantha** *Chaowasku*

*Hubera* Chaowasku

**Miliusa** *Lesch. ex A. DC.*

*Hyalostemma* Wall., *Saccopetalum* Benn.

**Orophea** *Blume*

*Mezzettiopsis* Ridl.

**Trivalvaria** (*Miq*.) *Miq.*

**Marsypopetalum** *Scheff.*

**Pseuduvaria** *Miq.*

*Craibella* R. M. K. Saunders, Y. C. F. Su & Chalermglin, *Oreomitra* Diels, *Petalolophus* K. Schum.

**Popowia** *Endl.*

**Polyalthia** *Blume*

*Haplostichanthus* F. Muell., *Papualthia* Diels, *Sphaerothalamus* Hook. f.

Annonoideae Raf.

Bocageeae Endl.

**Mkilua** *Verdc.*

**Hornschuchia** *Nees*

*Mosenodendron* R. E. Fr.

**Bocagea** *A. St.-Hil*.

**Trigynaea** *Schltdl.*

**Froesiodendron** *R. E. Fr.*

**Porcelia** *Ruiz & Pav.*

**Cardiopetalum** *Schltdl.*

*Stormia* S. Moore

**Cymbopetalum** *Benth.*

Xylopieae Endl.

**Artabotrys** *R. Br.*

*Ropalopetalum* Griff.

**Xylopia** *L.*

*Coelocline* A. DC., *Habzelia* A. DC., *Krockeria* Necker, *Parabotrys* Müll., *Parartabotrys* Miq., *Patonia* Wight, *Pseudannona* Saff., *Unona* L. f., *Waria* Aubl., *Xylopiastrum* Roberty, *Xylopicron* Adans., *Xylopicrum* P. Browne

Duguetieae Chatrou & R. M. K. Saunders

**Pseudartabotrys** *Pellegr.*

**Letestudoxa** *Pellegr.*

**Duckeanthus** *R. E. Fr.*

**Fusaea** (*Baill.*) *Saff.*

**Duguetia** *A. St.-Hil.*

*Aberemoa* Aubl., *Alcmene* Urb., *Geanthemum* (R. E. Fr.) Saff., *Pachypodanthium* Engl. & Diels

Guatterieae Hook. f. & Thomson

**Guatteria** *Ruiz & Pav.*

*Guatteriella* R. E. Fr., *Guatteriopsis* R. E. Fr., *Heteropetalum* Benth.

Annoneae Endl.

**Anonidium** *Engl. & Diels*

**Boutiquea** *Le Thomas*

**Neostenanthera** *Exell*

*Stenanthera* Engl. & Diels

**Goniothalamus** *Hook. f. & Thomson*

*Atrutegia* Bedd., *Beccariodendron* Warb., *Richella* A. Gray

**Diclinanona** *Diels*

**Disepalum** *Hook. f.*

*Enicosanthellum* Bân

**Asimina** *Adans.*

*Deeringothamnus* Small, *Orchidocarpum* Michx., *Pityothamnus* Small

**Annona** *L.*

*Guanabanus* Mill., *Raimondia* Saff., *Rollinia* A. St.-Hil., *Rolliniopsis* Saff.

Monodoreae Baill.

**Ophrypetalum** *Diels*

**Sanrafaelia** *Verdc.*

**Mischogyne** *Exell*

**Uvariodendron** (*Engl. & Diels*) *R. E. Fr.*

**Monocyclanthus** *Keay*

**Uvariopsis** *Engl.*

*Dennettia* Baker f., *Tetrastemma* Diels, *Thonnera* De Wild.

**Asteranthe** *Engl. & Diels*

*Asteranthopsis* Kuntze

**Hexalobus** *A. DC.*

**Uvariastrum** *Engl.*

**Monodora** *Dunal*

**Isolona** *Engl.*

Uvarieae Hook. f. & Thomson

**Uvaria** *L.*

*Anomianthus* Zoll., *Armenteria* Thouars ex Baill., *Balonga* Le Thomas, *Cyathostemma* Griff., *Dasoclema* J. Sinclair, *Ellipeia* Hook. f. & Thomson, *Ellipeiopsis* R. E. Fr., *Marenteria* Thouars, *Melodorum* Lour., *Narum* Adans., *Naruma* Raf., *Pyragma* Noronha, *Rauwenhoffia* Scheff., *Tetrapetalum* Miq., *Uva* Kuntze, *Uvariella* Ridl.

**Dielsiothamnus** *R. E. Fr.*

**Pyramidanthe** *Miq.*

**Mitrella** *Miq.*

**Fissistigma** *Griff.*

**Afroguatteria** *Boutique*

**Toussaintia** *Boutique*

**Cleistochlamys** *Oliv.*

**Sphaerocoryne** (*Boerl.*) *Scheff. ex Ridl.*

**Friesodielsia** *Steenis*

*Oxymitra* (Blume) Hook. f. & Thomson, *Schefferomitra* Diels

**Desmos** *Lour.*

**Dasymaschalon** (*Hook. f. & Thomson*) *Dalla Torre & Harms*

*Pelticalyx* Griff.

**Monanthotaxis** *Baill.*

*Atopostema* Boutique, *Clathrospermum* Planch. ex Benth., *Enneastemon* Exell, *Exellia* Boutique, *Gilbertiella* Boutique
